# CALPHAD-Based Multi-Objective Composition Screening of P91-Type Steel with Explainable Machine Learning

**DOI:** 10.3390/ma19214694

**Published:** 2026-11-04

**Authors:** Yinghu Wang, Luhan Tu, Enuo Wang, Zhendong Sheng, Xiaowen Cai, Hengyi Zhang, Lingxi Song

**Affiliations:** 1 Chengdu Advanced Metal Materials Industrial Technology Research Institute Co., Ltd., Chengdu 610300, China; 2 National Center for Materials Service Safety, University of Science and Technology Beijing, Beijing 100083, China

**Keywords:** P91 steel, CALPHAD, machine learning, SHAP, phase stability, multi-objective composition screening

## Abstract

The thermodynamic competition among multiple precipitate phases and its coupling with solidification behavior in P91-type steel remain insufficiently explored across multicomponent composition spaces, limiting the systematic identification of favorable alloy compositions. To address this gap, a framework integrating high-throughput CALPHAD calculations, explainable machine learning, and multi-objective screening was developed. A full-factorial design of 26,244 compositions yielded 19,838 complete datasets after excluding invalid calculations. The equilibrium mole fractions of M_23_C_6_, MX, AlN, Z phase, and Laves phase at 600 °C, together with the Scheil solidification interval, were investigated. Random Forest, XGBoost, and LightGBM models were evaluated, and SHAP analysis was used to interpret the composition-dependent predictions of thermodynamic responses. C was identified as the dominant contributor to the calculated M_23_C_6_ fraction and to the Scheil solidification interval, whereas N, Nb, and V strongly influenced the calculated MX fraction. Within the investigated composition space, increasing N was associated with a lower equilibrium MX fraction but higher AlN and Z-phase fractions, indicating competition among N-containing precipitates under the adopted equilibrium conditions. Direct Pareto screening of the CALPHAD results identified 812 non-dominated compositions. Subsequent TOPSIS evaluation favored C = 0.12, Mo = 0.85, Nb = 0.10, and N = 0.03 wt.% under the selected criteria. Relative to the base P91 composition, the highest-ranked candidate exhibited reductions of 39.99% and 98.30% in the Z-phase and Laves-phase fractions, respectively, together with a 12.93% increase in the M_23_C_6_ fraction. However, its Scheil solidification interval increased by 23.36 °C, revealing a trade-off between precipitation-related objectives and solidification behavior. The selected composition therefore represents a thermodynamically favorable candidate within the investigated discrete design space and under the adopted screening criteria, rather than a universally optimal composition for P91 steel.

## 1. Introduction

P91 steel is one of the most representative 9Cr–1Mo–V–Nb heat-resistant steels and has been widely used in high-temperature pressure-bearing components. The conventional heat-treatment practice involves normalizing at approximately 1040–1080 °C, followed by cooling and tempering at approximately 730–800 °C to obtain a tempered martensitic microstructure. This microstructure provides elevated-temperature strength through the combined contributions of solid-solution strengthening, dislocation strengthening, and precipitation strengthening [1]. Following normalization and tempering, P91 steel typically develops a tempered martensitic microstructure, and its high-temperature strength is provided by the combined effects of solid-solution strengthening, dislocation strengthening, and precipitation strengthening [2]. The development of a tempered martensitic microstructure is closely associated with austenite stability and its transformation during cooling. Alloying elements such as C and Ni influence austenite stability and martensitic transformation temperatures, thereby affecting microstructural evolution during heat treatment [3]. In addition to chemical composition, processing conditions and kinetic factors play important roles in phase evolution, as highlighted by studies on cryogenic-cycling-induced phase separation in metallic glasses [4]. Meanwhile, mechanical loading and environmental conditions can also affect the service behavior of steels, with stress-dependent passivation in alkaline environments providing an example of such interactions [5]. M_23_C_6_ carbides are mainly distributed along prior-austenite grain boundaries and martensitic lath, block, and packet boundaries, whereas fine MX carbonitrides are primarily located within the laths and effectively pin subgrain boundaries and dislocations [6,7,8,9,10]. During long-term exposure at elevated temperatures, recovery of the martensitic substructure and the formation, coarsening, and transformation of precipitates progressively alter the microstructural stability. Therefore, the evolution of precipitates is considered an important factor governing the long-term high-temperature performance of P91 steel [11]. After more than 100,000 h of service near 600 °C, M_23_C_6_ and MX precipitates can still be observed, together with the formation of Laves phase and a small amount of modified Z phase, indicating that this temperature range is representative of long-term service conditions [8,12].

The precipitates formed in P91 steel play different roles in microstructural stability and strengthening. Fine MX carbonitrides exhibit relatively slow coarsening and are important for maintaining precipitation strengthening during long-term service. M_23_C_6_ carbides help stabilize lath and subgrain boundaries, but their strengthening effect gradually decreases as they coarsen during prolonged high-temperature exposure [12,13]. Long-term service may also promote the formation of secondary phases such as Laves phase and Z phase [14,15]. Precipitation of Laves phase consumes Mo from the matrix and, together with subsequent particle coarsening, may reduce solid-solution strengthening. Modified Z phase, Cr(V,Nb)N, can form at the expense of the original MX precipitates during long-term exposure, thereby changing the contribution of MX to precipitation strengthening [13,16,17,18,19]. The Al content can also influence the competition among N-containing precipitates. Previous studies have shown that increasing Al promotes AlN formation and consumes N that would otherwise participate in the formation of VN-type MX, leading to a decrease in the MX fraction [20,21]. Thus, alloying elements such as C, N, V, Nb, Mo, and Al do not independently control individual precipitates; instead, they jointly affect the stability of multiple phases through elemental partitioning and competition among phases. For the compositional design of P91 steel, optimization based on a single precipitate is therefore insufficient to simultaneously maintain MX and M_23_C_6_ while controlling the formation of Z phase, AlN, and Laves phase.

Experimental studies can directly characterize the microstructural evolution of P91 steel during heat treatment and long-term service. However, systematically evaluating the coupled effects of multiple alloying elements on the stability of several precipitate phases generally requires considerable experimental effort. CALPHAD provides an alternative means of calculating phase equilibria and solidification behavior in multicomponent systems based on thermodynamic databases [22]. Earlier studies combined Thermo-Calc calculations with experimental characterization to investigate the evolution of M_23_C_6_, MX, and Laves phases in P91 steel as a function of temperature and thermomechanical history [23]. Rojas et al. used computational thermodynamics to assist the design of 9%Cr heat-resistant steels and examined the effects of compositional changes on the evolution of M_23_C_6_, MX, and Laves phases [24]. Smith et al. further applied CALPHAD to study the thermodynamic stability of M_23_C_6_, MX, and Z phase in Grade 91 steel, showing that variations in alloying elements can markedly alter the stability ranges of these secondary phases [25]. Nevertheless, most previous studies have focused on specific compositions, a limited number of compositional variables, or the stability of individual phases. When several alloying elements and multiple phase-stability criteria are considered simultaneously, the relationship between composition and thermodynamic response becomes increasingly multidimensional.

With the development of high-throughput computation and data-driven methods, machine learning has increasingly been used to model nonlinear relationships between alloy composition and thermodynamic or microstructural characteristics, and to assist the exploration of large compositional spaces [26,27,28]. Combining CALPHAD data with machine-learning models also enables the contributions of individual alloying elements to different outputs to be examined using feature-importance analysis and SHAP. More recently, CALPHAD, machine learning, and multi-objective optimization have been combined for composition design in steels, providing an additional means of addressing alloy-design problems involving several competing objectives [29]. In addition to precipitate stability during high-temperature service, solidification behavior is an important consideration in the compositional design of P91-type steel. Alloying elements can influence both the equilibrium stability of precipitates and the solidification temperature interval, with the latter reflecting aspects of solute redistribution and potentially relating to solidification-related defects during manufacturing. Consequently, compositions with favorable precipitate stability may not necessarily exhibit equally favorable solidification characteristics, highlighting the need to consider both aspects in alloy screening. Although CALPHAD, machine learning, and multi-objective optimization have been integrated in previous alloy-design studies, their combined application to the competing thermodynamic responses of multiple precipitate phases and solidification behavior in P91-type steel remains relatively limited. To address this gap, the present study combines high-throughput CALPHAD calculations, interpretable machine learning, and multi-objective screening to examine composition-dependent phase competition and the trade-offs between precipitate stability and solidification characteristics.

The present study aims to establish a thermodynamics-informed composition-screening framework for P91-type steel by integrating high-throughput CALPHAD calculations, interpretable machine learning, and multi-objective evaluation. Specifically, the objectives are to (i) characterize the effects of alloying composition on the equilibrium stability of M_23_C_6_, MX, AlN, Z phase, and Laves phase at 600 °C and on the Scheil solidification interval; (ii) evaluate the ability of machine-learning models to reproduce these composition-dependent thermodynamic responses and identify the key elemental contributions using SHAP analysis; and (iii) examine the trade-offs among the selected thermodynamic criteria through Pareto screening and TOPSIS evaluation to identify representative candidate compositions within the investigated design space.

## 2. Methods

### 2.1. Composition Design and High-Throughput Thermodynamic Calculations

The present study employed a computational composition-design approach without experimental chemical composition measurements. Nine alloying elements, namely C, Mn, Cr, Ni, Mo, V, Nb, N, and Al, were selected as independent compositional variables, with Fe taken as the balance. The investigated composition levels were predefined for the full-factorial design rather than obtained from experimentally characterized steel specimens. As summarized in Table 1, Cr was assigned four levels, while each of the remaining eight elements was assigned three levels, resulting in 26,244 designed compositions. All elemental contents were expressed in wt.% and used as inputs for the subsequent CALPHAD calculations. The composition-screening space was constructed using nine independently varied alloying elements to investigate their combined effects on the selected thermodynamic descriptors within a manageable full-factorial design. Si was not included as an independent variable in the present composition design, although its influence on phase stability and oxidation-related behavior remains relevant to broader alloy-design considerations. Equilibrium phase-fraction data were obtained for 26,050 compositions, while Scheil solidification data were available for 25,887 compositions, all of which could be matched with the corresponding equilibrium records. After excluding 21 records with anomalous MX branch identification and applying the descriptor-completeness criteria, 19,838 compositions were retained for subsequent machine-learning analysis and multi-objective screening. The data-cleaning procedures and exclusion statistics are detailed in Section 2.3.

The investigated composition ranges were selected to represent a constrained P91-type alloy-design space while allowing systematic variations in the principal elements affecting precipitate stability and solidification behavior. C influences carbide and carbonitride formation, whereas V and Nb are important MX-forming elements. N contributes to the stability of N-containing precipitates, including MX, AlN, and Z phase, while Al competes for N through AlN formation. Cr and Mo influence the stability of Cr-rich carbides and secondary phases, particularly M_23_C_6_ and Laves phase, whereas Mn and Ni can modify phase equilibria through their effects on matrix stability and elemental partitioning. The full-factorial design allows these elements to vary independently as compositional inputs, providing combinations through which their coupled thermodynamic responses can be examined. The resulting machine-learning models were developed to describe these responses within the investigated composition ranges rather than to extrapolate to arbitrary P91 steel compositions.

Thermodynamic calculations were performed using Thermo-Calc 2021b, with high-throughput calculations implemented through TC-Python 1.4. The TCFE11 thermodynamic database was used. CALPHAD-based calculations have been widely applied to alloy composition design. In the present work, the equilibrium phase fractions were obtained from thermodynamic calculations conducted over a temperature range of 300–1600 °C. Occasional convergence failures occurred during the high-throughput TC-Python calculations; ultimately, 26,050 equilibrium phase-fraction datasets and 25,887 Scheil datasets were successfully obtained. The subsequent modeling and analysis were based on these thermodynamic calculation results.

### 2.2. Thermodynamic Feature Extraction

The equilibrium phase fractions were evaluated at 600 °C, a representative temperature relevant to the elevated-temperature service conditions of P91-type steel. At this temperature, the relative thermodynamic stability of M_23_C_6_, MX, AlN, Z phase, and Laves phase provides useful insight into precipitate competition and alloying-element partitioning. As summarized in Table 2, the equilibrium mole fractions of these five phases at 600 °C and the Scheil solidification interval were selected as the six thermodynamic descriptors. The input compositions were expressed in wt.%, whereas the equilibrium phase fractions were dimensionless mole fractions. Together, these descriptors provide a consistent basis for evaluating composition-dependent phase stability and solidification characteristics within the investigated alloy system. The Scheil solidification calculations were conducted through TC-Python using the standard Scheil–Gulliver approach without additional user-defined diffusion settings. Under this approximation, the liquid is assumed to be completely mixed, local thermodynamic equilibrium is maintained at the solid–liquid interface, and solid-state back diffusion is neglected. C and N were not explicitly specified as fast-diffusing elements, and their redistribution during solidification was therefore governed by the conventional Scheil assumptions. The solidification interval was defined as the temperature difference between solid mole fractions of 0.01 and 0.99 and was used as a comparative descriptor of solidification behavior across the investigated compositions.

The original phase labels were first parsed, with the elemental compositions of the corresponding phase families checked to avoid possible label ambiguity in the high-throughput thermodynamic calculations. For the phase fractions at 600 °C, the corresponding value was taken directly when a valid record was available at this temperature. Otherwise, linear interpolation was performed between adjacent valid data points:

(1) $$f_{p}\left(600\right)=f_{p}\left(T_{a}\right)+\frac{600-T_{a}}{T_{b}-T_{a}}\left[f_{p}\left(T_{b}\right)-f_{p}\left(T_{a}\right)\right]\;\;\;T_{a}<600<T_{b}$$

Here, *p* denotes the phase branch, and *T_a_* and *T_b_* are the temperatures used to support the interpolation, with an interval not exceeding 20 °C. The individual MX branches were interpolated separately to 600 °C and then summed.

For the Scheil calculation results, reverse linear interpolation was performed between adjacent points along the solidification path to determine the temperatures corresponding to the specified solid mole fractions. The Scheil solidification interval was defined as:

(2) $$\Delta T_{\mathrm{Scheil}}=T_{f_{s}=0.01}-T_{f_{s}=0.99}$$

If the specified threshold was not reached, multiple distinct intersections were present, or a non-unique plateau occurred, the result was retained as a missing value. No extrapolation was performed.

### 2.3. Dataset Construction and Cleaning

An initial dataset was constructed from 25,887 composition records for which both phase-fraction and Scheil calculation results were available. An MX candidate branch was considered anomalous if its maximum mole fraction over the entire temperature range exceeded 0.1, or if its mole fraction remained above 0.01 at temperatures higher than 1200 °C. Based on these numerical screening criteria, 21 compositions were excluded, leaving 25,866 thermodynamic calculation records. When a phase curve did not contain a data point exactly at 600 °C, the endpoint around the target temperature and up to three neighboring points were examined. The phase fraction at 600 °C was assigned a value of zero only when the endpoint mole fraction fell within the specified range, at least two valid points were available, the phase fractions along the curve were non-decreasing within the prescribed tolerance, and the temperature interval between adjacent points did not exceed 20 °C. Such cases were recorded as zero-boundary values. Curves containing conflicting phase fractions at the same temperature, invalid fraction values, or otherwise indeterminate behavior were excluded. For MX, when the identity of the relevant subphases could not be reliably determined, the partial sum of the remaining branches was not used as a substitute for the total MX fraction. After data cleaning, 19,838 compositions with complete and valid feature values were retained for machine-learning analysis and multi-objective screening. Pearson correlation coefficients were then calculated to examine the linear correlations between the nine compositional inputs and the six output variables. When a phase class was not exported for a composition and was treated as absent according to the predefined data-processing rule, its value was assigned as zero and retained with a separate provenance label. The reduction from 25,866 thermodynamic records to 19,838 complete datasets was primarily associated with the inability to reliably extract one or more target descriptors under the predefined phase-identification, interpolation, and boundary-assignment criteria. Among the 6028 excluded records, 5098 contained missing MX descriptors only, 623 contained missing AlN descriptors only, 259 contained missing MX and AlN descriptors, 33 contained missing MX and Laves-phase descriptors, and 15 contained missing Laves-phase descriptors only. Most missing MX descriptors were associated with insufficient valid data-point resolution near the target temperature or the absence of an unambiguous phase boundary. These records were excluded rather than assigned uncertain values, thereby preserving the consistency of the six-output dataset used for subsequent modeling and screening.

### 2.4. Construction and Evaluation of Machine Learning Models

Considering the potentially complex nonlinear relationships between alloy composition and thermodynamic responses, as well as interactions among alloying elements, three representative tree-based ensemble regression algorithms were compared in this study. Random forest regression (RF) is a bagging-based ensemble method, whereas extreme gradient boosting (XGBoost) and Light Gradient Boosting Machine (LightGBM) are gradient-boosting algorithms. These models have been widely used for composition–property modeling and alloy design [30,31]. For each of the six outputs, a separate regression model was developed using the nine compositional inputs, giving a total of 18 models. The same train–test split was used for all targets and algorithms, with a ratio of 4:1, corresponding to 15,870 training samples and 3968 test samples. Because tree-based models are relatively insensitive to the scale of the input variables, no input standardization was applied. The machine-learning models were developed to characterize the nonlinear relationships between alloy composition and the CALPHAD-derived thermodynamic descriptors and to provide a basis for interpreting the contributions of individual alloying elements. In the present framework, machine learning served as a response-modeling and interpretation tool, whereas the subsequent Pareto screening was conducted directly using the calculated thermodynamic data. This distinction allowed the composition screening to remain based on the original CALPHAD results while using the trained models to investigate compositional effects and interactions.

For each model, 20 hyperparameter combinations were evaluated using three-fold cross-validation, and the mean cross-validation score was used for hyperparameter selection. The tuned hyperparameters included the number of trees, tree depth, minimum number of samples per leaf, and, where applicable, the learning rate, row and feature subsampling ratios, and regularization parameters. After the optimal hyperparameters were selected, each model was refitted using the full training set and then evaluated on the test set. In addition, five-fold cross-validation was performed on the training set using the selected hyperparameters, and the mean and sample standard deviation of (R^2^) and RMSE were reported. The selected hyperparameters for all 18 regression models are provided in Supplementary Table S1. The same training–test split and hyperparameter-search procedure were applied across the six thermodynamic descriptors to ensure a consistent basis for model comparison.

For RF, the candidate numbers of trees were 150, 250, 400, and 600. For XGBoost and LightGBM, the corresponding values were 250, 400, 600, and 850, with candidate learning rates of 0.025, 0.05, 0.08, and 0.12. Fixed random seeds were used for data splitting, cross-validation shuffling, and randomized hyperparameter search to ensure reproducibility. The models were implemented using scikit-learn 1.6.1, XGBoost 3.0.4, and LightGBM 4.6.0 in Python 3.13.5.

Model performance on the training and test sets was evaluated using the coefficient of determination (R^2^), root mean square error (RMSE), and mean absolute error (MAE):

(3) $$R^{2}=1-\frac{\sum_{i=1}^{n}(y_{i}-{\hat{y}}_{i}{)}^{2}}{\sum_{i=1}^{n}(y_{i}-\bar{y}{)}^{2}}$$

(4) $$\mathrm{RMSE}=\sqrt{\frac{1}{n}\sum_{i=1}^{n}(y_{i}-{\hat{y}}_{i}{)}^{2}}$$

(5) $$\mathrm{MAE}=\frac{1}{n}\sum_{i=1}^{n}\left|y_{i}-{\hat{y}}_{i}\right|$$

Here, *y_i_* denotes the CALPHAD-derived value, ${\hat{y}}_{i}$ is the model prediction, and $\bar{y}$ is the mean target value of the corresponding evaluation set. For MX and AlN, the evaluation metrics were also calculated for the nonzero subsets of the test data. Residual analysis was further performed for MX, with the residual defined as the predicted value minus the CALPHAD-derived value. For each target, the three algorithms were compared based on the test set *R*^2^ and the selected model was used for the subsequent interpretation analysis.

### 2.5. SHAP Interpretability Analysis

For the best-performing model, SHAP values were calculated using TreeExplainer for all 3968 samples in the fixed test set. Here, *ϕ_ij_* denotes the contribution of the *j*th feature to the prediction of sample *i*, and *I_j_* represents the corresponding global feature importance. SHAP summary plots were generated by ranking the features according to *I_j_*. To compare relative importance across different targets, the *I_j_* values were normalized by the maximum value within each target. The element with the largest absolute Pearson correlation coefficient was also compared with the top-ranked feature identified by SHAP. According to the SHAP formulation, a model prediction can be expressed as the sum of the baseline value and the contributions of individual features [32,33]:

(6) $${\hat{y}}_{i}=\phi_{0}+\sum_{j=1}^{9}\phi_{\mathrm{ij}}$$

(7) $$I_{j}=\frac{1}{n}\sum_{i=1}^{n}\left|\phi_{\mathrm{ij}}\right|$$

### 2.6. Multi-Objective Component Screening Based on Pareto and TOPSIS

A discrete screening was performed using the 19,838 thermodynamic calculation results, with the objectives of maximizing *f*_MX,600_ while minimizing *f*_AlN,600_, *f_Z_*_,600_, *f*_Laves,600_ and Δ*T*_Scheil_. A composition A was considered to dominate composition B for all objectives and strictly better for at least one objective. All non-dominated compositions were retained [34].

The resulting 812 Pareto solutions were further ranked using TOPSIS [35]. Let *x_ij_* denote the value of the *j*th objective for the *i*th solution. Vector normalization and normalized weights were then applied as follows:

(8) $$r_{\mathrm{ij}}=\frac{x_{\mathrm{ij}}}{\sqrt{\sum_{i=1}^{m}x_{\mathrm{ij}}^{2}}}$$

*v_ij_* = *w_j_r_ij_* (9)

(10) $$\sum_{j=1}^{5}w_{j}=1$$

MX was treated as the benefit-type objective, for which the positive ideal value was defined as the maximum. The remaining objectives were treated as cost-type criteria, with their positive ideal values defined as the minima; the corresponding negative ideal values were defined in the opposite manner. The Euclidean distances from each solution to the positive and negative ideal solutions, $D_{i}^{+}$ and $D_{i}^{-}$, were then calculated, and the top 20 solutions were retained according to the closeness coefficient *S_i_* in descending order:

(11) $$D_{i}^{\pm }=\sqrt{\sum_{j=1}^{5}(v_{\mathrm{ij}}-v_{j}^{\pm }{)}^{2}}$$

(12) $$S_{i}=\frac{D_{i}^{-}}{D_{i}^{+}+D_{i}^{-}}$$

As shown in Table 3, four sets of weights were selected, and the degree of overlap of the top 20, the ranking of representative components, and the core element window were compared. For each weighting scheme, the TOPSIS scores and rankings of the Pareto solutions were recalculated.

## 3. Results and Discussion

### 3.1. Thermodynamic Feature Distributions and Correlation Analysis

**Figure 1:**
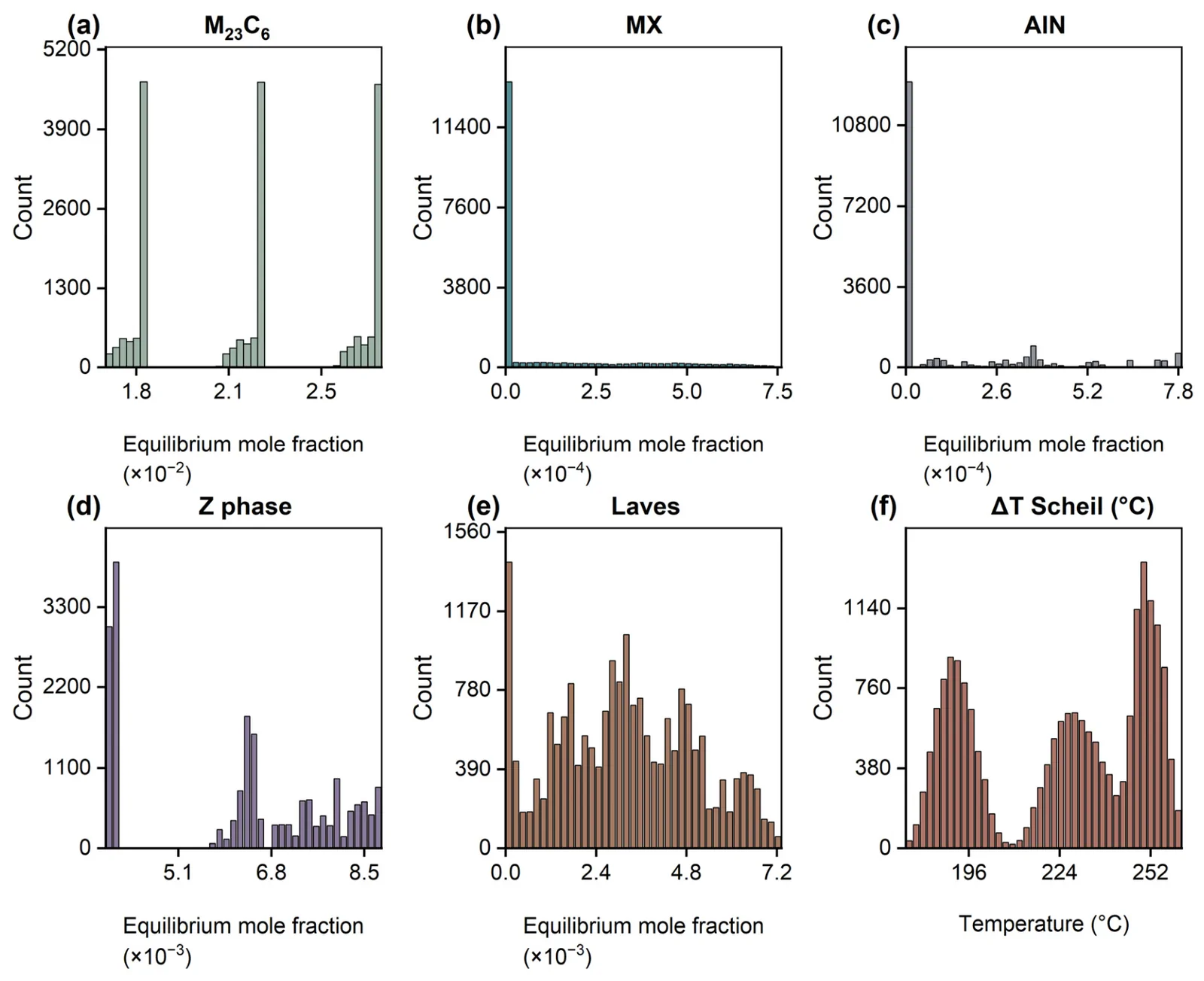
Distributions of the six output variables: (**a**) equilibrium mole fraction of M_23_C_6_ at 600 °C; (**b**) equilibrium mole fraction of MX at 600 °C; (**c**) equilibrium mole fraction of AlN at 600 °C; (**d**) equilibrium mole fraction of Z phase at 600 °C; (**e**) equilibrium mole fraction of Laves phase at 600 °C; and (**f**) Scheil solidification interval.

Figure 1 shows the distributions of the six output variables obtained from the 19,838 complete thermodynamic calculation records. At 600 °C, the equilibrium mole fraction of M_23_C_6_ ranged from 0.01635 to 0.02678, with a mean value of 0.02206. The mean mole fractions of the Z phase and Laves phase were 0.00610 and 0.00318, respectively. The distributions of MX and AlN were concentrated mainly at zero and in the low-mole-fraction range, with maximum values of 7.608 × 10^−4^ and 7.892 × 10^−4^, respectively. Zero-valued cases accounted for 5.04% of the Laves phase data, whereas no zero values were observed for the M_23_C_6_ or Z phase. The six descriptors exhibited different degrees of skewness and multimodality. In particular, the grouped distribution of M_23_C_6_ may be related to the discrete C levels used in the composition design.

**Figure 2:**
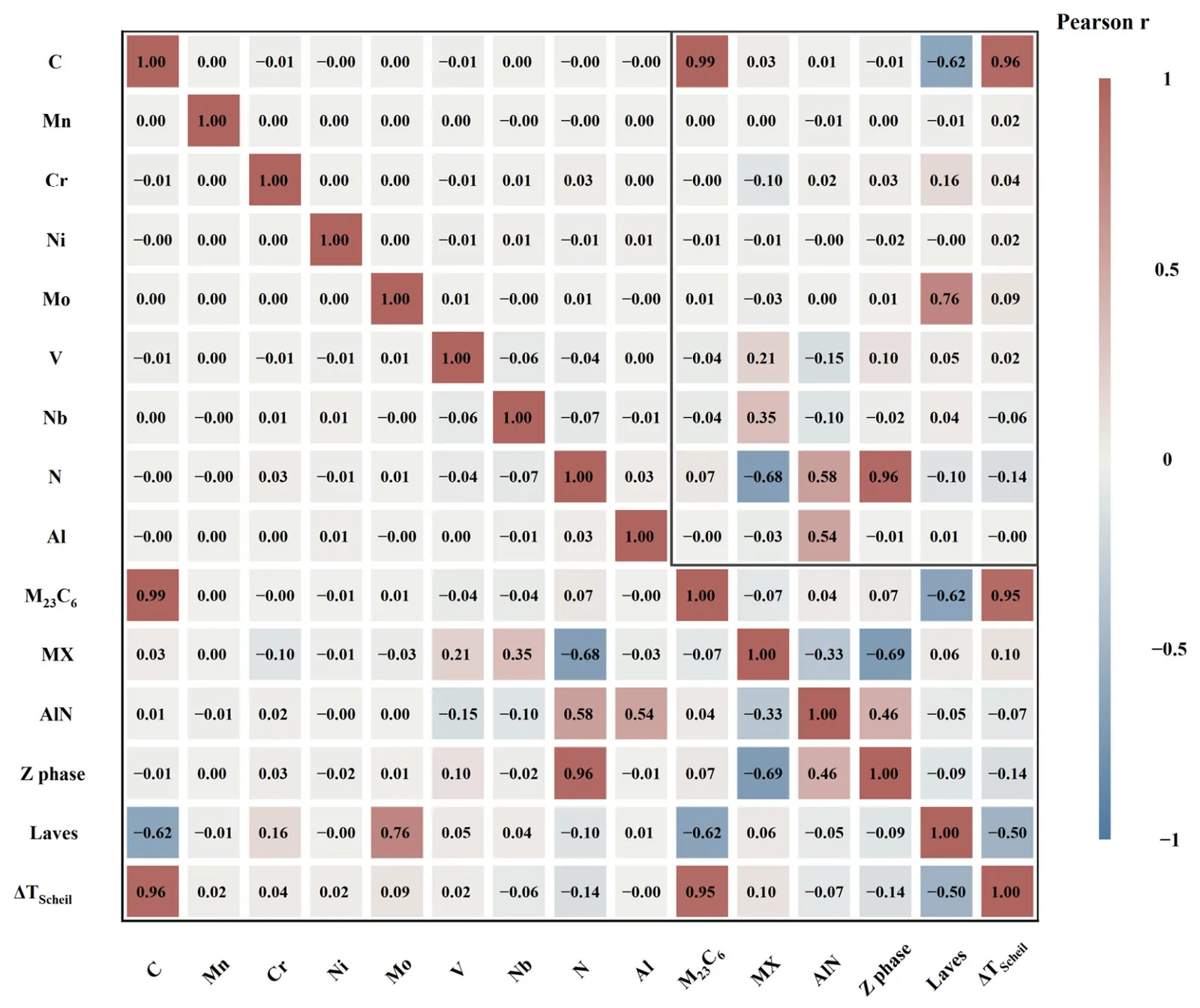
Pearson correlation heatmap. Colors and numerical values indicate the correlation coefficients, and the bold-outlined region highlights the correlations between the input variables and output variables.

The Pearson correlation matrix between the nine elemental inputs and the six output variables is shown in Figure 2. C exhibited a strong positive correlation with the M_23_C_6_ mole fraction, with a correlation coefficient of 0.99. When grouped by C content, the mean M_23_C_6_ mole fractions were 0.01761, 0.02206, and 0.02648 at C levels of 0.08, 0.10, and 0.12 wt.%, respectively. The differences among these group means correspond to the three main distribution regions observed for M_23_C_6_ in Figure 1a. In P91 steel, M_23_C_6_ is one of the major carbide phases of interest, and variations in C content can affect both the availability of carbide-forming elements and the equilibrium phase constitution. Therefore, within the investigated composition range and under equilibrium conditions at 600 °C, the M_23_C_6_ mole fraction shows an approximately linear overall relationship with C content.

The Pearson correlation coefficient between C and the Scheil solidification interval was 0.96, indicating a strong positive correlation. At C contents of 0.08, 0.10, and 0.12 wt.%, the mean solidification intervals were 191.81, 229.76, and 251.88 °C, respectively, giving a difference of 60.07 °C between the highest and lowest C levels. Thus, a higher C content was associated not only with a higher equilibrium fraction of M_23_C_6_ but also with a wider Scheil solidification interval. This trend may be related to the influence of C on solid–liquid phase equilibria and solute partitioning during solidification in multicomponent steels [36,37].

N showed different correlation trends with the N-containing outputs. Its correlation coefficient with Z phase was 0.96, whereas that with MX was −0.68, indicating that increasing N was associated with an increase in Z phase and a decrease in MX. At N contents of 0.03, 0.05, and 0.07 wt.%, the mean mole fractions of Z phase were 0.003910, 0.006305, and 0.007772, respectively, while the corresponding mean MX fractions were 3.044 × 10^−4^, 2.270 × 10^−6^, and 0. At 0.07 wt.% N, all MX values were zero, whereas the proportions of zero-valued MX cases at 0.03 and 0.05 wt.% N were 10.19% and 94.92%, respectively. The correlation coefficients of Nb and V with MX were 0.349 and 0.211, respectively, showing an overall positive relationship between Nb and V contents and the equilibrium MX fraction within the investigated composition range. Previous studies have shown that MX precipitates in 9Cr–1Mo–V–Nb steels are mainly Nb- and V-rich carbonitrides, whose composition and stability are jointly influenced by Nb, V, C, and N [37]. Because both MX and Z phase involve Nb, V, and N, and Z-phase formation may be accompanied by the consumption of pre-existing MX carbonitrides, a potential competition in phase stability may exist between them [17]. AlN showed positive correlations with both N and Al, with correlation coefficients of 0.58 and 0.54, respectively. When grouped by N content, the mean AlN mole fraction was 0 at 0.03 wt.% N and increased to 5.260 × 10^−5^ and 2.979 × 10^−4^ at 0.05 and 0.07 wt.% N, respectively.

Mo showed a positive correlation with the equilibrium mole fraction of the Laves phase, with a correlation coefficient of 0.76. At Mo contents of 0.85, 0.95, and 1.05 wt.%, the mean Laves phase fractions were 0.001474, 0.003166, and 0.004920, respectively. All 1000 zero-valued Laves cases occurred in the 0.85 wt.% Mo group, indicating the important role of Mo in Laves phase formation. Meanwhile, C was negatively correlated with the Laves phase fraction, with a correlation coefficient of −0.62, suggesting that the Laves phase fraction was also related to C content. Changes in C content may alter the carbide fraction and be accompanied by redistribution of other alloying elements among the equilibrium phases, thereby affecting the formation of other phases.

Although the nine alloying elements were independently assigned in the original full-factorial design, their effects on the calculated thermodynamic descriptors are not necessarily independent. The observed relationships reflect the combined influence of phase equilibria, elemental partitioning, and competition among precipitates. Moreover, the subsequent exclusion of incomplete calculation records may alter the statistical distribution of the retained compositions. Accordingly, the correlation and machine-learning results are interpreted within the final analyzed dataset rather than as independent causal effects of individual alloying elements.

### 3.2. Comparison of Machine Learning Models

Table 4 compares the predictive performance of the three regression algorithms on the test set. LightGBM achieved the highest test R^2^ for all six targets. Except for MX, both RF and LightGBM yielded test R^2^ values above 0.99 for the other five targets, indicating that these descriptors could be fitted with high accuracy within the investigated discrete composition space. The differences among the models were more pronounced for MX, for which the test R^2^ values of RF, XGBoost, and LightGBM were 0.95742794, 0.89769468, and 0.95844524, respectively. Although LightGBM achieved the highest test R^2^ for all six descriptors, its advantage over RF was relatively small for MX. In particular, RF yielded a lower test MAE, whereas LightGBM showed slightly better R^2^ and RMSE values. Therefore, the model comparison should not be interpreted as demonstrating the general superiority of LightGBM over RF. LightGBM was selected primarily to maintain a consistent modeling and SHAP interpretation framework across the six targets.

**Figure 3:**
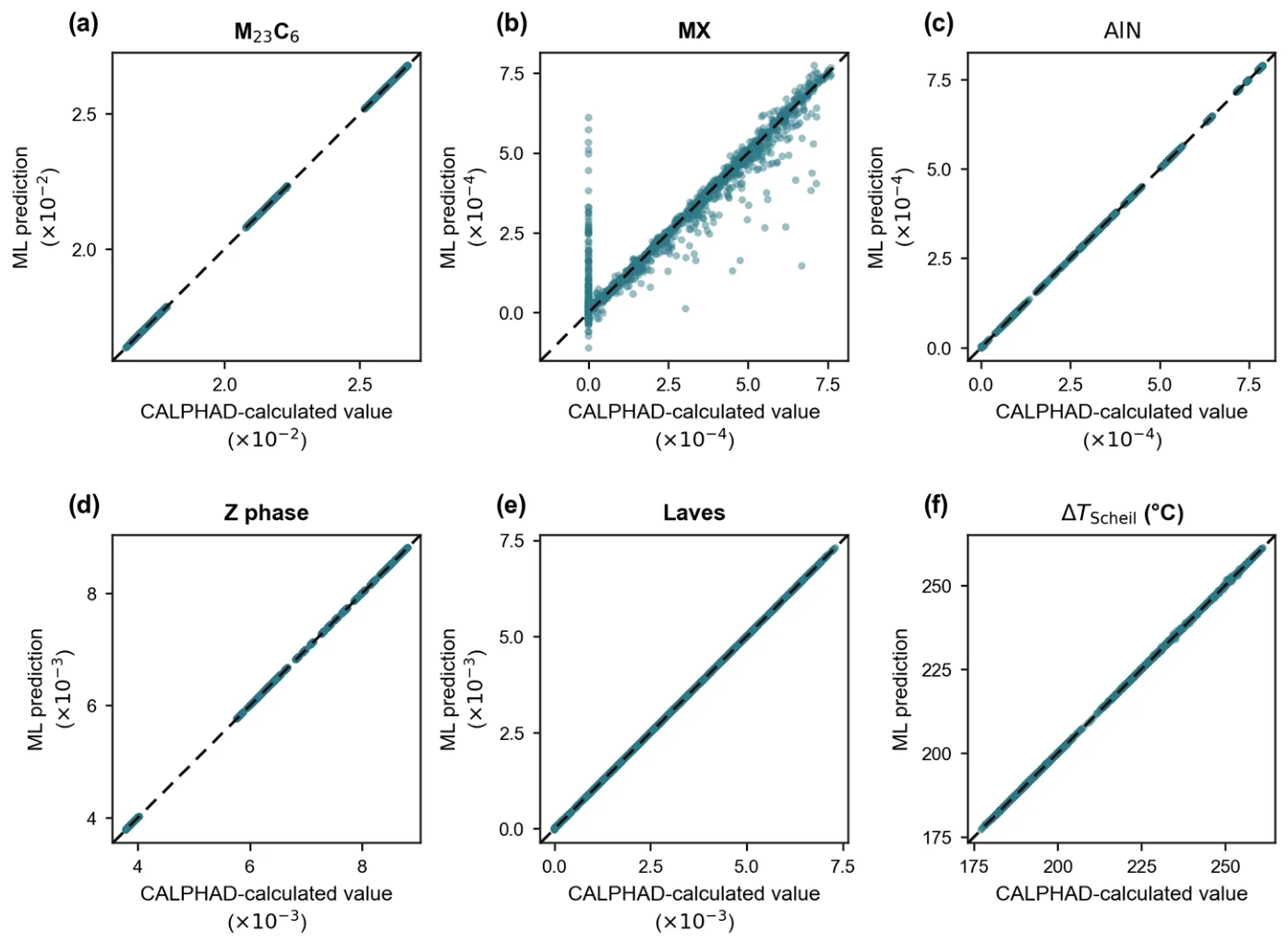
Comparison between the LightGBM predictions and CALPHAD-derived values on the test set: (**a**) equilibrium mole fraction of M_23_C_6_ at 600 °C; (**b**) equilibrium mole fraction of MX; (**c**) equilibrium mole fraction of AlN; (**d**) equilibrium mole fraction of Z phase; (**e**) equilibrium mole fraction of Laves phase; and (**f**) Scheil solidification interval.

Figure 3 compares the LightGBM predictions with the corresponding CALPHAD-calculated values. Except for MX, the test R^2^ values for the other five targets were all above 0.99, with most prediction points distributed close to the y = x reference line. The test RMSE values for M_23_C_6_ and Z phase were 1.6187 × 10^−6^ and 5.3047 × 10^−7^, respectively. For the Scheil solidification interval, the test RMSE and MAE were 0.125842 °C and 0.079232 °C, respectively, consistent with the limited scatter observed in Figure 3f.

The MX predictions showed greater scatter around the reference line. The LightGBM model achieved an R^2^ of 0.98482201 on the training set and 0.95844524 on the test set, giving a difference of 0.02638 and indicating some disparity between the training and test fits. In addition, the five-fold cross-validation R^2^ for MX on the training set was 0.96139915 ± 0.00475384, which was close to the test-set result. This suggests that the hold-out evaluation was generally consistent with the cross-validation performance, although the larger scatter observed for MX still requires further interpretation. When only the nonzero MX samples in the test set were considered, LightGBM achieved an R^2^ of 0.96293847, with RMSE and MAE values of 4.0310 × 10^−5^ and 1.7806 × 10^−5^, respectively. These values were close to those obtained for the full test set, indicating that the overall prediction performance was not strongly affected by the large number of zero-valued samples. For comparison, the 1445 nonzero AlN samples in the test set yielded an R^2^ of 0.99999785 and an RMSE of 3.3295 × 10^−7^. Thus, although both MX and AlN contained a substantial number of zero values, their prediction errors differed markedly.

The MX residual was defined as the predicted value minus the CALPHAD-calculated value. In the test set, 51 samples with a true value of zero were predicted to have values above 1 × 10^−4^, while 43 samples with nonzero true values showed absolute relative errors greater than 50%. The sample with the largest absolute residual had a CALPHAD-calculated value of 0 and a predicted value of 6.1072 × 10^−4^. The positive predictions for zero-valued samples and the underestimation of some nonzero values in Figure 3b indicate that the transition between MX absence and presence is an important source of model-fitting difficulty, although the current residual analysis does not allow the errors to be attributed to a single cause. The 50 samples with the largest absolute residuals all occurred at N = 0.03 wt.%, and their mean Nb content was 0.0964 wt.%, higher than the overall test-set mean of 0.07965 wt.%. This indicates that the larger prediction errors were compositionally biased within the present dataset.

The above performance primarily reflects the ability of the models to reproduce the thermodynamic calculation results within the investigated composition space. A high R^2^ therefore indicates high predictive accuracy for the CALPHAD-calculated values in this domain and should not be interpreted as an independent measure of predictive accuracy for experimentally measured precipitate fractions.

The high prediction accuracy obtained for most descriptors primarily reflects the ability of the regression models to reproduce deterministic CALPHAD-derived responses within the sampled composition space. Since the training and test sets were drawn from the same discrete full-factorial design, the reported R^2^ values do not necessarily establish predictive reliability for intermediate compositions, alloys outside the investigated ranges, or experimentally measured phase fractions. The MX descriptor presents an additional modeling challenge because its response includes a transition between zero and nonzero equilibrium fractions. A two-stage classification–regression approach, in which phase presence is identified before predicting its nonzero fraction, may provide a useful alternative for representing this behavior. Further validation using independently calculated compositions outside the original sampling grid, together with experimental measurements where available, would help assess the broader generalization capability of these models.

### 3.3. SHAP Analysis

**Figure 4:**
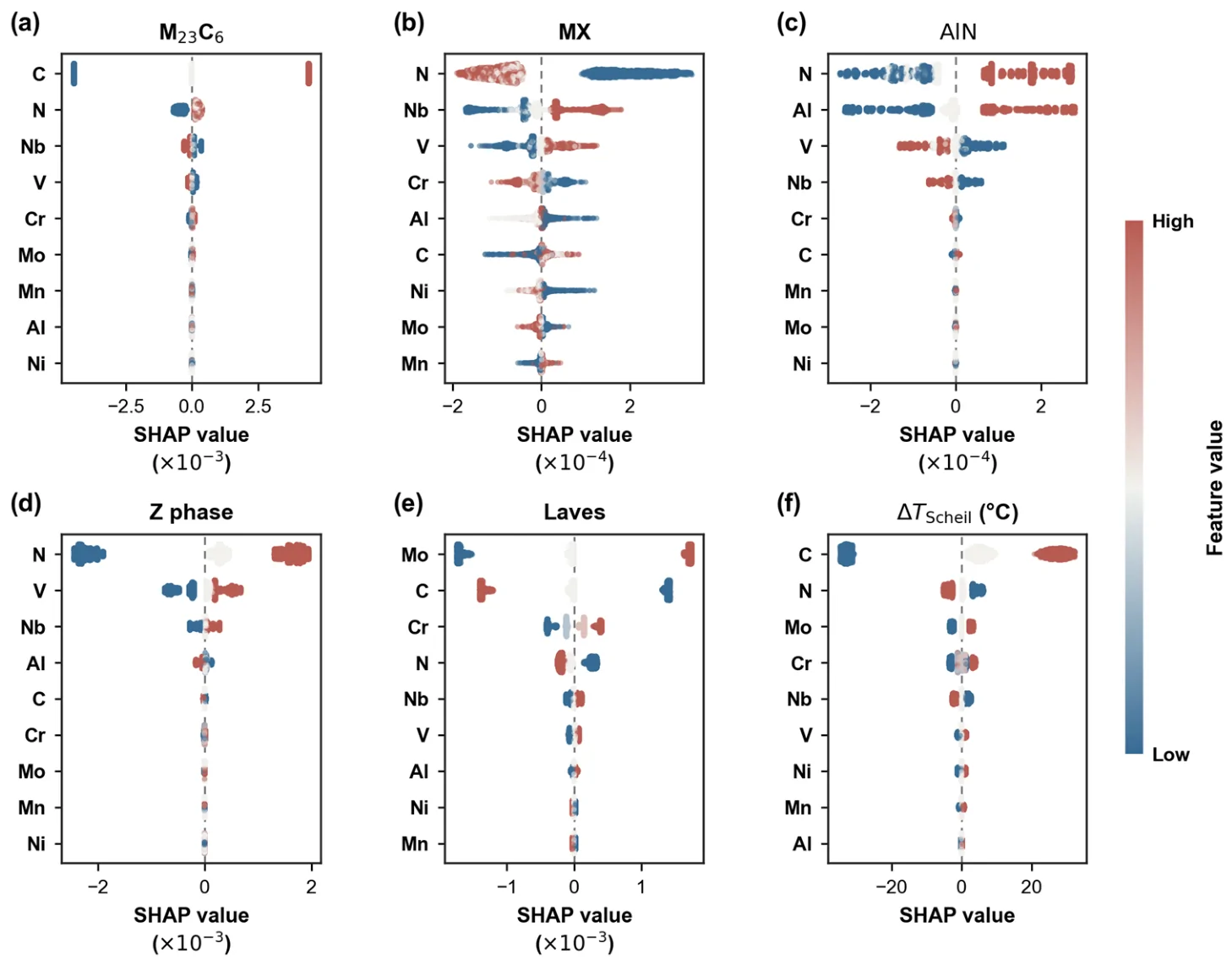
SHAP analysis of the LightGBM models: (**a**) equilibrium mole fraction of M23C6 at 600 °C; (**b**) equilibrium mole fraction of MX; (**c**) equilibrium mole fraction of AlN; (**d**) equilibrium mole fraction of Z phase; (**e**) equilibrium mole fraction of Laves phase; and (**f**) Scheil solidification interval.

Figure 4 presents the SHAP results for the LightGBM models based on the 3968 test samples. For M_23_C_6_, the most influential elements were C, N, and Nb, while C, N, and Mo showed the largest contributions to the Scheil solidification interval. For MX, AlN, and Z phase, N was the most influential element, followed by Nb and V for MX, Al and V for AlN, and V and Nb for Z phase. The Laves phase was mainly influenced by Mo, C, and Cr. For each output, the element ranked first by SHAP was consistent with the element showing the largest absolute Pearson correlation coefficient. This agreement indicates that the dominant linear relationships observed in the original dataset were also reflected in the model-based feature contribution ranking.

**Figure 5:**
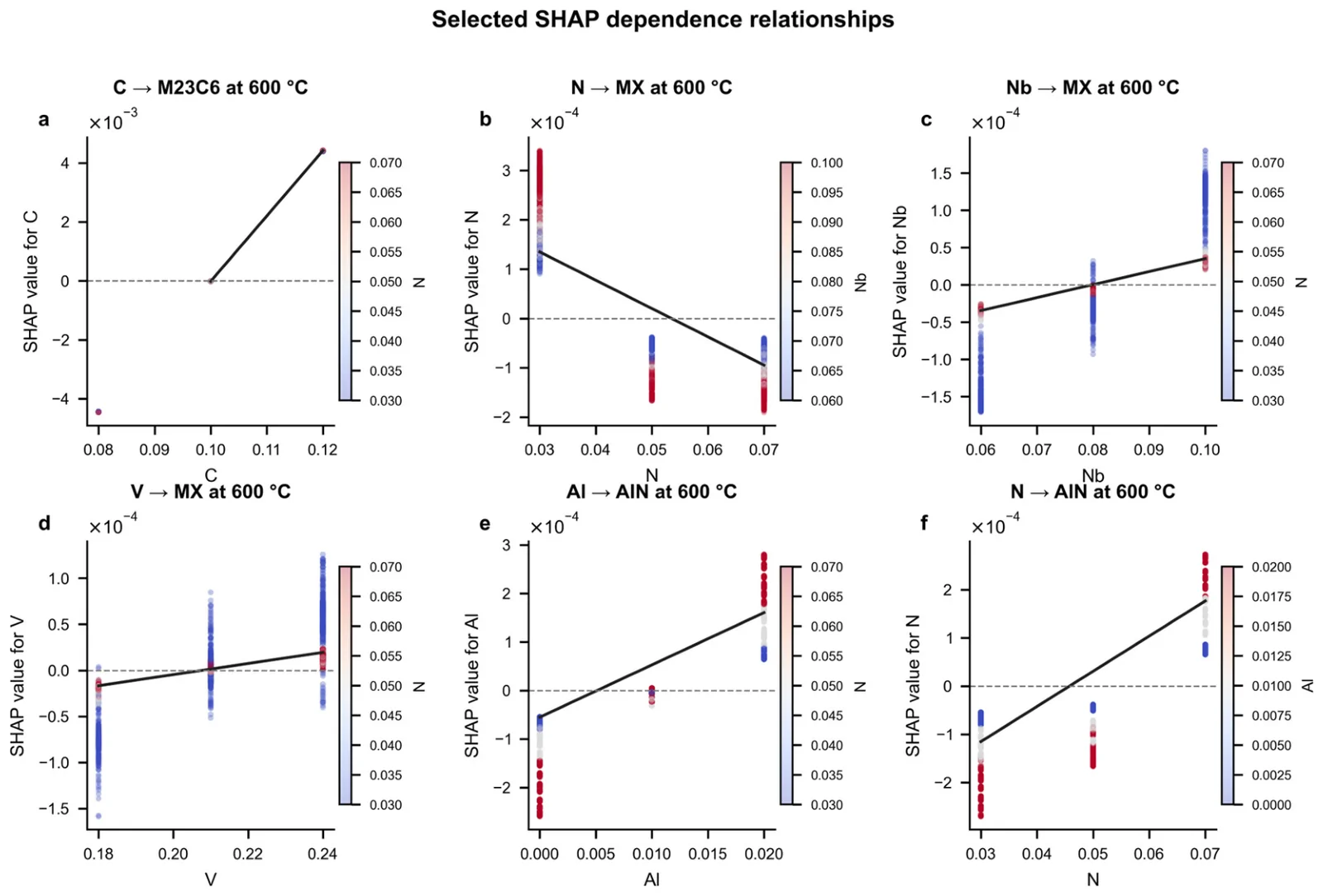
SHAP dependence plots showing the contributions of key elements to the LightGBM outputs: (**a**) C–M_23_C_6_; (**b**) N–MX; (**c**) Nb–MX; (**d**) V–MX; (**e**) Al–AlN; and (**f**) N–AlN. The gray dashed line indicates zero contribution, and the black solid line represents the median.

C had the largest influence on both M_23_C_6_ and the Scheil solidification interval, with mean absolute SHAP values of 2.9495 × 10^−3^ and 21.7534 °C, respectively. The corresponding mean absolute SHAP values of N were 2.8864 × 10^−4^ and 3.2322 °C, both lower than those of C. As shown in Figure 5a, the SHAP contribution of C to M_23_C_6_ changed from negative to positive as the C level increased. The Spearman correlation coefficient between C content and its SHAP contribution was 0.94, and the corresponding coefficient in the solidification-interval model was also 0.94. This is consistent with the previously observed positive correlations of C with both the M_23_C_6_ fraction and the Scheil solidification interval. Increasing the C content was therefore associated with a higher equilibrium carbide fraction and a wider Scheil solidification interval. In the MX model, the mean absolute SHAP values of N, Nb, and V were 1.3034 × 10^−4^, 5.2010 × 10^−5^, and 2.5416 × 10^−5^, respectively. The Spearman correlation coefficient between N content and its SHAP contribution to MX was −0.79, whereas the corresponding coefficients for Nb and V were both 0.91. Figure 5b shows that lower N levels were mainly associated with positive contributions, while higher N levels were mainly associated with negative contributions. In Figure 5c,d, the contributions of Nb and V generally shifted toward more positive values as their contents increased. However, the contribution magnitude at a given Nb or V level was not constant. For samples with N = 0.03 wt.%, the mean Nb contribution at Nb = 0.10 wt.% was 1.2665 × 10^−4^, whereas the corresponding value at N = 0.07 wt.% was 3.2643 × 10^−5^. At lower Nb levels, the low-N group also showed larger negative contributions, and a similar trend was observed for V. These results are closely related to nitride formation and indicate that the effects of Nb and V cannot be interpreted independently of the N level. As carbonitride-forming elements, Nb and V showed an overall positive contribution to the MX model, whereas the opposite contribution of N to MX compared with other N-containing phases suggests that equilibrium phase stability and elemental competition may both be involved in this response.

In the AlN model, the mean absolute SHAP values of N and Al were 1.3551 × 10^−4^ and 1.0064 × 10^−4^, respectively. The Spearman correlation coefficients between their contents and corresponding SHAP contributions were 0.85 and 0.94. As shown in Figure 5e, the contribution of Al changed from negative to positive as the Al level increased, while samples with higher N contents showed larger positive and negative contribution magnitudes. In Figure 5f, the SHAP contribution of N generally increased with N content, with a larger variation observed at higher Al levels. For example, at Al = 0.02 wt.%, the mean Al contributions for the N = 0.03 and 0.07 wt.% groups were 7.4765 × 10^−5^ and 2.3448 × 10^−4^, respectively. This trend is consistent with the fact that AlN formation depends on the availability of both Al and N.

In the Z-phase model, N had the largest mean absolute SHAP value of 1.5542 × 10^−3^, followed by V and Nb with values of 2.7490 × 10^−4^ and 9.0519 × 10^−5^, respectively. The Spearman correlation coefficient between N content and its SHAP contribution to Z phase was 0.931, and the contribution generally shifted in the positive direction as the N level increased. Considering the positive contribution of N to AlN and its negative contribution to MX, the same alloying element can exert different effects on different N-containing phases. The influence of dataset completeness on the interpretation of the N–MX relationship was also examined. Although the original full-factorial design contained equal numbers of compositions at N levels of 0.03, 0.05, and 0.07 wt.%, the corresponding retention rates in the final dataset were 79.23%, 50.23%, and 97.31%, respectively. The lower retention rate at N = 0.05 wt.% was mainly associated with difficulties in extracting the MX descriptor near its presence–absence transition. Within the retained dataset, the proportion of nonzero MX values decreased from 89.81% at N = 0.03 wt.% to 5.08% at N = 0.05 wt.% and 0% at N = 0.07 wt.%. The distribution of N levels in the SHAP test set closely followed that of the complete dataset, and N remained the most influential feature in the MX model. These observations support the interpretation of the N–MX relationship within the retained data while indicating that the unequal retention rates should be considered when extending the conclusions to the original composition space.

The combined effects of V, Nb, C, and N on precipitate stability can be understood in terms of carbonitride formation and elemental partitioning. V and Nb are important MX-forming elements, while C and N occupy the interstitial sublattice of MX carbonitrides and influence their thermodynamic stability. Changes in the C/N balance may therefore affect the relative stability of carbide- and nitride-rich precipitates. Meanwhile, N also participates in the formation of competing phases, particularly AlN and Cr(V,Nb)N-type Z phase, depending on the availability of Al, Cr, V, and Nb. Consequently, the influence of V and Nb on the equilibrium MX fraction is closely related to the accompanying C and N contents and the stability of other precipitates. This interpretation is consistent with the positive SHAP contributions of V and Nb to MX and the contrasting contributions of N to MX, AlN, and Z phase. The negative contribution of N to MX in the present dataset thus reflects the combined equilibrium response of the investigated alloy system rather than an intrinsic suppression of MX formation by N [13,24]. Similar thermodynamic competition has been discussed in previous studies on Grade 91 steel. Smith et al. investigated the stability of M_23_C_6_, MX, and Z phase using computational thermodynamics, while Sawada et al. reported changes in the MX precipitate population associated with Z-phase formation during prolonged creep exposure [25,38]. These findings provide a metallurgical context for the phase competition identified in the present study. Nevertheless, the SHAP results describe composition-dependent equilibrium responses, whereas the experimental observations reflect microstructural evolution under specific exposure conditions.

In the Laves phase model, the mean absolute SHAP values of Mo and C were 1.1559 × 10^−3^ and 9.2852 × 10^−4^, respectively, with Cr showing the next largest influence. The Spearman correlation coefficient between Mo content and its SHAP contribution was 0.943. As shown in Figure 4e, higher Mo levels were mainly associated with positive contributions, whereas higher C levels were mainly associated with negative contributions. These trends are consistent with the positive Mo–Laves correlation and the negative C–Laves correlation identified from the Pearson analysis.

### 3.4. Pareto Frontier

Among the 19,838 compositions, five screening objectives were considered: increasing the equilibrium mole fraction of MX while reducing the fractions of AlN, Z phase, and Laves phase, as well as the Scheil solidification interval. The resulting non-dominated set contained 812 compositions. A comparison of the objective distributions for the full dataset and the Pareto set is provided in Table 5. The mean MX fraction increased, whereas the mean fractions of AlN, Z phase, and Laves phase decreased. The mean Scheil solidification interval increased slightly from 224.521 to 224.612 °C, while the median decreased from 229.299 to 226.970 °C.

**Figure 6:**
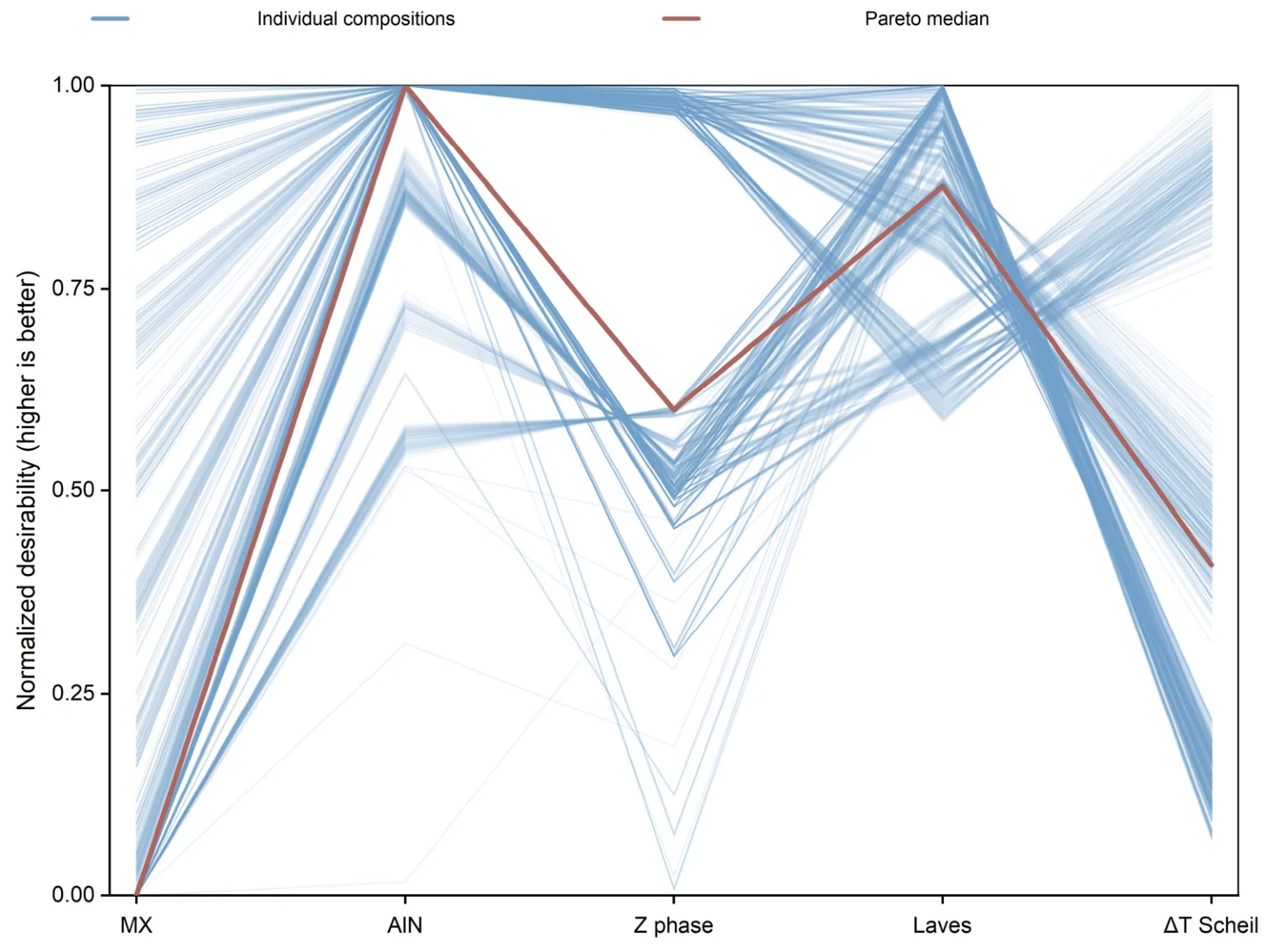
Normalized distributions of the five objectives for the 812 Pareto solutions. Blue lines represent individual compositions, while the red line indicates the median value for each objective. Higher values on the vertical axis correspond to more favorable directions according to the screening criteria.

Figure 6 shows the normalized distributions of the five objectives for the 812 Pareto compositions. Each objective was normalized using the value range of the complete dataset, with higher normalized values representing more favorable directions according to the screening criteria. The crossing of lines between different objectives indicates that increasing MX, reducing the fractions of the other phases, and narrowing the solidification interval cannot always be achieved simultaneously. Within the Pareto set, the MX fraction still ranged from 0 to 7.6077 × 10^−4^. Some solutions showed low AlN fractions but also low or zero MX fractions. Consequently, the non-dominated set does not provide a single uniquely preferred composition.

**Figure 7:**
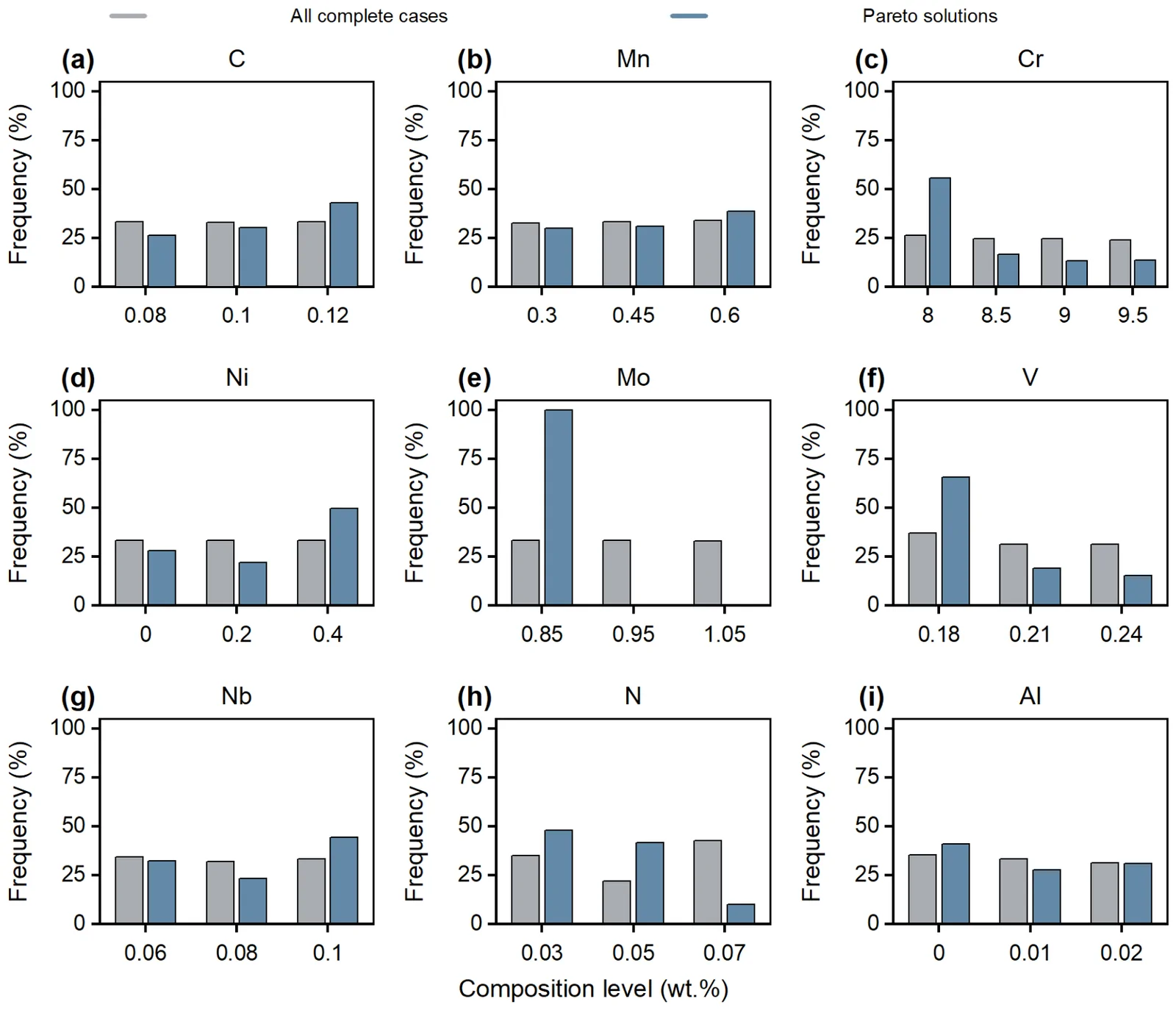
Comparison of elemental-level frequencies between the complete dataset and the 812 Pareto solutions: (**a**) C; (**b**) Mn; (**c**) Cr; (**d**) Ni; (**e**) Mo; (**f**) V; (**g**) Nb; (**h**) N; and (**i**) Al.

The frequencies of the discrete elemental levels are compared in Figure 7. The most pronounced concentration was observed for Mo: all 812 Pareto compositions contained Mo = 0.85 wt.%, whereas this level accounted for only 33.41% of the complete dataset. This preference for the lower Mo level is consistent with the previously observed positive correlation between Mo and the Laves phase fraction, as well as its positive model contribution. Under the present five-objective screening scheme, a lower Mo content therefore favored inclusion in the non-dominated set, with 0.85 wt.% representing the lower boundary of the investigated Mo range. The proportion of Cr = 8.00 wt.% increased from 26.48% to 55.91%, that of Ni = 0.40 wt.% from 33.41% to 49.88%, and that of Nb = 0.10 wt.% from 33.38% to 44.46%. These changes indicate that the Pareto set was enriched in compositions with relatively low Cr, high Ni, and high Nb levels.

Within the Pareto set, the numbers of compositions with N = 0.03, 0.05, and 0.07 wt.% were 391, 340, and 81, corresponding to 48.15%, 41.87%, and 9.98%, respectively. In the complete dataset, the corresponding proportions were 34.94%, 22.15%, and 42.91%. The fraction of the highest N level therefore decreased markedly, although the intermediate level still accounted for a substantial proportion of the Pareto set. Considering the previously identified relationships between N and the different N-containing phases, lower N levels were generally compatible with higher MX fractions and lower AlN and Z-phase fractions.

The C distribution did not shift systematically toward lower levels despite the Scheil solidification interval being treated as a cost-type objective. The proportion of C = 0.12 wt.% increased from 33.55% to 43.10%, whereas that of C = 0.08 wt.% decreased from 33.36% to 26.35%. Together with the absence of a decrease in the mean solidification interval, this indicates that the Pareto set still contains compositions that accept a wider solidification interval in exchange for improvements in other objectives.

The frequency trend of V differed from the single-objective tendency inferred from its positive contribution to MX. The proportion of V = 0.18 wt.% increased to 65.76% in the Pareto set, compared with 37.24% in the complete dataset, whereas that of V = 0.24 wt.% decreased from 31.27% to 15.27%. Thus, the non-dominated set as a whole showed a preference for lower V levels. The proportion of Al = 0 increased from 35.43% to 41.13%, indicating a moderate preference for lower Al, while the proportion of Mn = 0.60 wt.% increased only slightly from 34.07% to 38.92%.

Although M_23_C_6_ was not included as a direct objective in the multi-objective screening, its overall distribution was also examined. Its mean mole fraction changed from 0.022058 in the complete dataset to 0.022647 in the Pareto set, while the corresponding median values were 0.022277 and 0.022300. Therefore, no evident overall decrease in M_23_C_6_ was observed in the Pareto set.

### 3.5. TOPSIS-Based Candidate Composition Screening and Comprehensive Evaluation

Among the 812 Pareto solutions, an equal-weight TOPSIS evaluation was first performed using the five objectives. All of the top 20 compositions had C = 0.12, Ni = 0.40, Mo = 0.85, Nb = 0.10, and N = 0.03 wt.%, with each of these levels accounting for 100% of the Top 20. This indicates that the highest-ranked candidates were concentrated within a relatively narrow composition range. The preference for lower N and higher Nb is consistent with the previously identified contribution trends in the MX model, while the lower Mo level agrees with the direction for reducing the Laves descriptor. The higher C level, however, involves a trade-off between an increased M_23_C_6_ fraction and a wider solidification interval. Among the Top 20 solutions, 13 compositions contained V = 0.24 wt.% and 7 contained V = 0.21 wt.%, whereas none had V = 0.18 wt.%. This differs from the overall Pareto set, which showed a preference for lower V. The non-dominated set retains a broad range of trade-offs, whereas the TOPSIS ranking under the present weighting scheme favors candidates associated with higher MX fractions.

The ranking results obtained using the four weighting schemes are summarized in Table 6. The top-ranked composition under the equal-weight scheme remained first under Scheme A, Scheme B, in which the MX weight was doubled, and Scheme C, in which the relative weights of AlN, Z phase, and Laves phase were each increased to 1.5. Under Scheme D, where the solidification-interval weight was doubled, this composition dropped to sixth place, while the preferred composition under Scheme D moved to first. The Top 20 sets from Schemes B, C, and D shared 15, 20, and 17 compositions, respectively, with the original equal-weight Top 20, and 15 compositions were common to all four Top 20 sets. Across all four schemes, the core levels of C = 0.12, Mo = 0.85, Nb = 0.10, and N = 0.03 wt.% were retained. This indicates that the preferred composition window was relatively stable, although the detailed ranking still depended on the weighting of the objectives. To further evaluate the robustness of the TOPSIS rankings, an additional sensitivity analysis was conducted using 100 randomly generated positive weight combinations sampled from a Dirichlet distribution, while retaining the original 812 Pareto solutions and the same five screening objectives. Candidate 1 ranked first under 49 weighting combinations, while Candidate 2 ranked first under 9 combinations; both candidates appeared among the top 20 under 73 combinations. A total of 22 different compositions occupied the first position, indicating that the ranking of individual candidates is sensitive to the assigned objective weights. Nevertheless, the principal compositional preferences remained relatively consistent. Across the 2000 top-20 occurrences, Mo = 0.85, Nb = 0.10, N = 0.03, and C = 0.12 wt.% accounted for 100%, 96.15%, 96.05%, and 73.40%, respectively. These findings suggest that, although the highest-ranked composition may vary with the weighting scheme, the present screening identifies a relatively consistent group of candidate compositions within the investigated discrete design space. The sensitivity analysis therefore supports the interpretation of the results as a candidate composition window rather than a uniquely optimal alloy composition.

M_23_C_6_ was not included among the five direct optimization objectives. A retention criterion was therefore introduced at 90% of the median value of the complete dataset, corresponding to an equilibrium mole fraction of at least 0.0200490 at 600 °C. The Pareto set contained 391, 340, and 81 compositions at N levels of 0.03, 0.05, and 0.07 wt.%, respectively; therefore, the observed compositional preferences should be interpreted within the retained complete dataset, particularly given the unequal sample retention rates across the three N levels. Of the 812 Pareto solutions, 598 compositions satisfied this criterion, accounting for 73.65% of the total. All of the original equal-weight Top 20 solutions were retained, and the members of the original Top 5 also remained unchanged, although their internal ranking changed after applying the constraint. 

The composition at the center of the investigated design space was selected from the database as the reference composition. Its composition and those of five representative candidates are listed in Table 7, and their six output variables are summarized in Table 8. The central reference composition was selected as a consistent computational baseline within the investigated design space, rather than as an experimentally characterized P91 specimen. Compared with the reference composition, the top-ranked candidate under the equal-weight scheme increased the MX fraction from 0 to 7.6060 × 10^−4^. The Z-phase and Laves-phase fractions decreased by 39.99% and 98.30%, respectively, while the M_23_C_6_ fraction increased by 12.93%. The AlN fraction was recorded as zero for both compositions, although the origins of the zero values were different. The Scheil solidification interval increased from 232.216 to 255.580 °C, corresponding to an increase of 23.364 °C and a 10.06% deterioration relative to the objective of reducing the solidification interval. Thus, this candidate improved several phase-related objectives at the expense of a wider solidification interval.

Candidate 1 represents the high-ranking solution jointly favored by Schemes A, B, and C. Candidate 2 represents the preferred solution when greater weight was assigned to the solidification interval; compared with Candidate 1, it had a slightly lower MX fraction and a slightly narrower solidification interval. Candidate 3 ranked 2nd, 3rd, 3rd, and 3rd under Schemes A, B, C, and D, respectively, providing an intermediate candidate with relatively stable rankings across the four weighting schemes. Candidate 4 ranked 18th under Scheme D and had the narrowest solidification interval among the five selected candidates, at 251.704 °C. However, its MX fraction decreased to 7.0361 × 10^−4^, while the Laves phase fraction increased to 3.8978 × 10^−4^, reflecting a trade-off that places greater emphasis on the solidification interval. Candidate 5 ranked 18th under Scheme A, with an MX fraction of 6.7242 × 10^−4^, while maintaining relatively low Z-phase and Laves-phase fractions. The equal-weight TOPSIS scores of Candidates 1 and 2 were 0.956625 and 0.956364, respectively.

**Figure 8:**
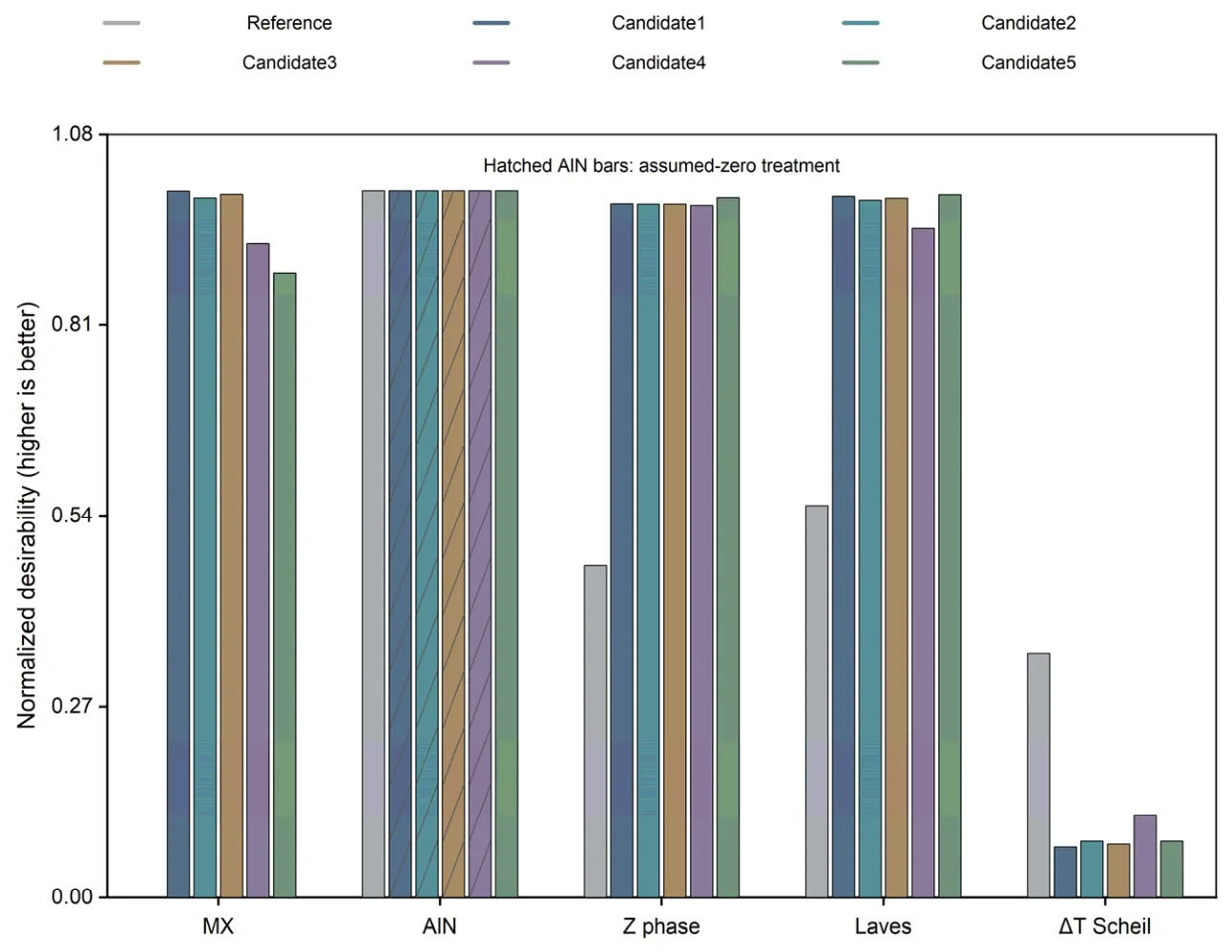
Normalized comparison of the central reference composition and the five representative candidates.

Figure 8 compares the normalized five-objective performance of the reference composition and the five representative candidates, with higher normalized values indicating more favorable performance according to the corresponding objective direction. The candidate compositions generally performed better in terms of MX, Z phase, and Laves phase, whereas their normalized solidification-interval performance was lower than that of the reference composition. 

The compositional preferences identified in this study should be interpreted in the context of the selected thermodynamic objectives and the investigated design space. The preference for Mo = 0.85 wt.% is primarily associated with minimizing the equilibrium Laves-phase fraction, although Mo also contributes to solid-solution strengthening of the martensitic matrix. Similarly, the selection of Cr = 8.0 wt.% in several highly ranked candidates reflects the adopted screening criteria rather than an assessment of oxidation resistance. Several preferred compositions also lie at the upper boundaries of C, Nb, and Ni and the lower boundary of N, indicating that the identified composition window is influenced by the resolution and limits of the full-factorial design. In particular, the pronounced variation in the calculated MX fraction between N = 0.03 and 0.05 wt.% suggests sensitivity to local compositional changes, which may affect the relative ranking of candidates under realistic manufacturing tolerances. Therefore, the selected compositions represent favorable candidates within the present thermodynamic screening framework rather than continuously optimized alloys with experimentally established superior high-temperature performance. For practical alloy development, additional metallurgical and service-related considerations remain important. The effects of C and Ni on austenite stability and martensitic transformation behavior, as well as the potential formation of δ-ferrite during processing, can influence the development of the desired tempered martensitic microstructure. Moreover, oxidation and corrosion resistance, mechanical stress, and processing-dependent microstructural evolution may affect long-term service performance but are not explicitly represented by the current optimization objectives. The influence of stress–environment interactions on steel passivation, for example, has been demonstrated under aqueous alkaline conditions, although these findings should not be directly equated with oxidation behavior in high-temperature steam environments. Future extensions of the framework may incorporate additional performance-related constraints and local composition sensitivity analyses to support more comprehensive and robust alloy design.

### 3.6. Thermodynamic Characterization of the Selected Candidate Composition

**Figure 9:**
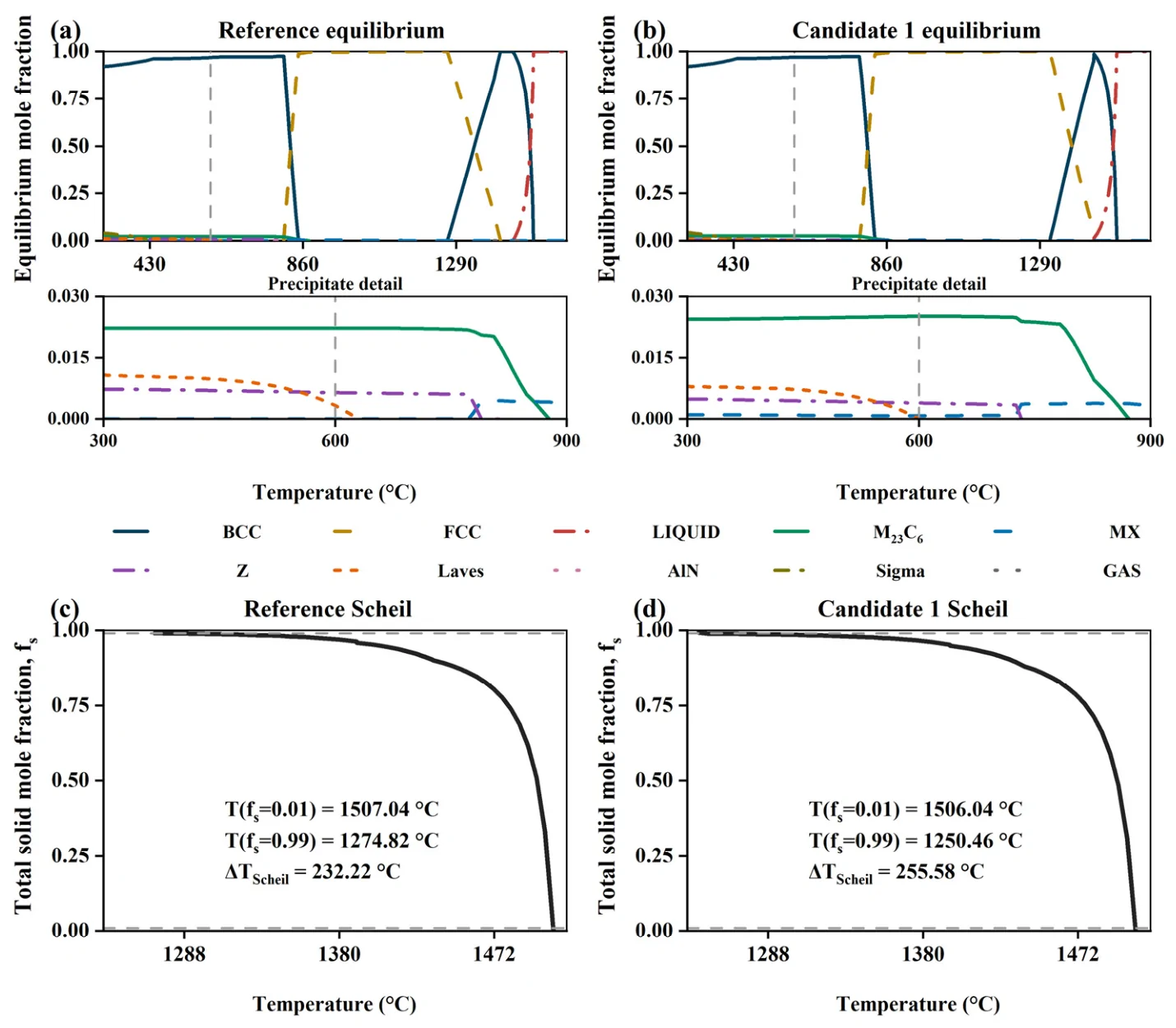
Thermodynamic comparison between the central reference composition and Candidate 1: (**a**) equilibrium phase evolution of the reference composition; (**b**) equilibrium phase evolution of Candidate 1; (**c**) Scheil solidification path of the reference composition; and (**d**) Scheil solidification path of Candidate 1.

Figure 9 compares the equilibrium phase evolution and Scheil solidification behavior of the central reference composition and Candidate 1. As shown in Figure 9a,b, the two compositions exhibit broadly similar phase-transition behavior at high temperatures, whereas clear differences are observed in the stability ranges and fractions of the low-temperature precipitate phases. At 600 °C, Candidate 1 shows a higher equilibrium mole fraction of M_23_C_6_ and a nonzero MX fraction, while the fractions of Z phase and Laves phase are lower than those of the reference composition, consistent with the preceding multi-objective screening results. Further comparison of the phase-stability ranges shows that the upper stability limit of the Z phase decreases from approximately 790.0 °C in the reference composition to about 733.1 °C in Candidate 1, while that of the Laves phase decreases from approximately 629.6 to 600.5 °C. This indicates that the lower Z-phase and Laves-phase fractions of Candidate 1 at 600 °C are accompanied by a contraction of their stability ranges toward lower temperatures. In contrast, the upper stability limit of M_23_C_6_ changes only slightly, decreasing from approximately 877.2 °C in the reference composition to about 872.4 °C in Candidate 1. Thus, the higher M_23_C_6_ fraction of Candidate 1 at 600 °C is not accompanied by a pronounced extension of its stability range. 

The phase-stability advantages observed for Candidate 1 at 600 °C do not persist over the entire temperature range. Candidate 1 has a nonzero equilibrium MX fraction at 600 °C, whereas the reference composition has zero MX at this temperature. However, as the temperature increases, the relative MX fractions of the two compositions change, indicating that the MX advantage of Candidate 1 near the screening temperature is strongly temperature dependent. A similar trend is observed for M_23_C_6_: Candidate 1 exhibits a higher fraction near 600 °C, but the difference between the two compositions gradually decreases as the temperature approaches the upper stability limit. Therefore, the advantages of Candidate 1 are mainly evident near the 600 °C temperature range considered in this study and should not be interpreted as higher MX and M_23_C_6_ stability over the entire temperature range.

Figure 9c,d show the Scheil solidification paths of the two compositions. Compared with the reference composition, the temperature corresponding to a solid fraction of f_s_ = 0.01 for Candidate 1 decreases by only about 1.0 °C, whereas the temperature corresponding to f_s_ = 0.99 decreases by approximately 24.36 °C. The widening of the solidification interval in Candidate 1 therefore mainly results from the extension of the high-solid-fraction stage toward lower temperatures rather than from a substantial shift in the onset of solidification. These results further show that, although Candidate 1 provides a more favorable combination of MX, Z phase, and Laves phase near 600 °C, this improvement is achieved at the expense of a wider non-equilibrium solidification interval, consistent with the multi-objective trade-off identified by the Pareto screening and TOPSIS evaluation.

To examine the thermodynamic competition between MX and Z phase, additional metastable equilibrium calculations were performed at 600 °C for the reference composition and Candidate 1 with Z phase suppressed. Under full equilibrium conditions, the calculated MX mole fractions were 0 and 7.6060 × 10^−4^, respectively, increasing to 4.2481 × 10^−3^ and 3.3811 × 10^−3^ when the Z phase was suppressed. The substantial increase in MX fraction indicates strong competition between these phases and provides a possible thermodynamic explanation for the retention of MX precipitates in service-exposed P91 steel. Importantly, suppressing the Z phase reverses the relative MX fractions of the two compositions, demonstrating that their ranking depends on the assumed equilibrium conditions. Accordingly, the original multi-objective screening remains a comparison under the specified full-equilibrium criteria, whereas the metastable calculations provide complementary insight into MX stability when Z-phase formation is restricted. The temperature dependence of Laves-phase stability was further examined using the equilibrium phase-evolution results between 550 and 650 °C. At 550 and 600 °C, the calculated Laves-phase mole fractions decreased from 6.789 × 10^−3^ to 3.262 × 10^−3^ for the reference composition and from 3.900 × 10^−3^ to 5.545 × 10^−5^ for Candidate 1. At 625 °C, the Laves phase remained stable only in the reference composition, with upper stability boundaries of approximately 629.6 °C and 600.5 °C for the reference composition and Candidate 1, respectively. These results indicate that Candidate 1 exhibits a lower equilibrium Laves-phase fraction over the temperature range in which the phase is stable, although the magnitude of the reduction varies considerably with temperature. Thus, the reported 98.30% reduction at 600 °C represents a temperature-specific comparison rather than a uniform improvement across the entire range. The metallurgical significance of these predictions can be considered in relation to previous studies of long-term microstructural evolution in P91 steel. Panait et al. identified M_23_C_6_, MX, Laves phase, and a small amount of modified Z phase in Grade 91 steel after 113,431 h of creep exposure at 600 °C [8,12]. Zhao et al. also reported Laves-phase precipitation and growth during prolonged aging of P91 steel at 625 °C, accompanied by changes in the carbide population [39]. These observations highlight the importance of precipitate stability for long-term service performance. M_23_C_6_ carbides contribute to the stabilization of martensitic lath and subgrain boundaries, while fine MX carbonitrides impede dislocation motion and help maintain precipitation strengthening. During prolonged thermal exposure, precipitate coarsening and changes in phase constitution may weaken these contributions. In particular, Z-phase formation can occur at the expense of fine MX precipitates, whereas Laves-phase precipitation can lead to Mo depletion from the matrix and affect solid-solution strengthening. Such microstructural changes have been associated with the degradation of creep resistance in P91 steels [40]. The present calculations provide a complementary thermodynamic perspective by comparing the relative stability of these phases across different compositions, while their actual evolution during service also depends on precipitation kinetics and thermal exposure history.

## 4. Conclusions

In this study, high-throughput CALPHAD calculations, interpretable machine learning, and multi-objective screening were combined to investigate composition-dependent precipitate stability and solidification behavior in P91-type steel. The main conclusions corresponding to the research objectives are summarized as follows.

(1) Analysis of 19,838 complete CALPHAD-derived records revealed distinct elemental influences on the five equilibrium phase fractions at 600 °C and the Scheil solidification interval. C exhibited strong positive correlations with the M_23_C_6_ fraction and solidification interval, while Mo was positively associated with the Laves-phase fraction. Under the adopted full-equilibrium conditions, N was positively associated with AlN and Z phase but negatively associated with MX, reflecting the competing thermodynamic responses of N-containing precipitates.
(2) Among the three evaluated algorithms, LightGBM achieved the highest test-set R^2^ values for all six thermodynamic descriptors, with an R^2^ of 0.95845 for MX and values exceeding 0.99997 for the remaining five outputs. SHAP analysis identified C as the dominant contributor to the M_23_C_6_ and solidification-interval models, whereas N, Nb, and V were the main contributors to the MX model. These results demonstrate the usefulness of interpretable machine learning for examining composition–thermodynamic response relationships within the investigated dataset.
(3) Pareto screening based directly on the CALPHAD results identified 812 non-dominated compositions. TOPSIS evaluation and weighting sensitivity analysis indicated a preference for C = 0.12, Mo = 0.85, Nb = 0.10, and N = 0.03 wt.% under the adopted screening criteria. Compared with the reference composition, the highest-ranked candidate under equal weighting exhibited reductions of 39.99% and 98.30% in the Z-phase and Laves-phase fractions, respectively, and a 12.93% increase in the M_23_C_6_ fraction. However, its Scheil solidification interval increased by 23.36 °C, demonstrating the trade-off between the selected objectives. The identified composition should therefore be regarded as a candidate solution within the investigated discrete composition space rather than a universally optimal P91 steel composition.

## Figures and Tables

**Table 1:** Predefined compositional levels used for the full-factorial CALPHAD screening (wt.%).

Element	Composition Levels (wt.%)	Number of Levels
C	0.08, 0.10, 0.12	3
Mn	0.30, 0.45, 0.60	3
Cr	8.00, 8.50, 9.00, 9.50	4
Ni	0.00, 0.20, 0.40	3
Mo	0.85, 0.95, 1.05	3
V	0.18, 0.21, 0.24	3
Nb	0.06, 0.08, 0.10	3
N	0.03, 0.05, 0.07	3
Al	0.00, 0.01, 0.02	3
Fe	Balance	-

**Table 2:** Selected six output variables and their definitions.

Output	Variable Name	Definition
*f*_M23C6,600_	f_M_23_C_6__600	Mole fraction of M_23_C_6_ at 600 °C
*f*_MX,600_	f_MX_600	Sum of the mole fractions of valid MX candidate branches at 600 °C
*f*_AlN,600_	f_AlN_600	Mole fraction of ALN_B4 at 600 °C
*f_Z_*_,600_	f_Z_600	Mole fraction of Z_PHASE at 600 °C
*f*_Laves,600_	f_Laves_600	Mole fraction of C14_LAVES at 600 °C
*Δ* *T*_Scheil_	DeltaT_Scheil	Temperature difference corresponding to an increase in the solid mole fraction from 0.01 to 0.99 (°C)

**Table 3:** Weighting schemes used for the TOPSIS sensitivity analysis (relative weights before normalization).

Scheme	MX	AlN	Z Phase	Laves	Scheil Solidification Interval
A	1	1	1	1	1
B	2	1	1	1	1
C	1	1.5	1.5	1.5	1
D	1	1	1	1	2

**Table 4:** Comparison of the test-set performance of the three regression models (For the five equilibrium phase-fraction outputs, the tabulated RMSE and MAE values should be multiplied by 10^−6^ to obtain the corresponding dimensionless errors. For the Scheil solidification interval, RMSE and MAE are reported directly in °C.

Output	Model	Test R^2^	Test RMSE	Test MAE
M_23_C_6_	RF	0.99999902	3.5995	1.9501
M_23_C_6_	XGBoost	0.99988592	38.880	26.455
M_23_C_6_	LightGBM	0.99999980	1.6187	0.64023
MX	RF	0.95742794	40.018	6.8652
MX	XGBoost	0.89769468	62.035	29.498
MX	LightGBM	0.95844524	39.537	11.015
AlN	RF	0.99998514	0.89886	0.35797
AlN	XGBoost	0.98838360	25.128	19.117
AlN	LightGBM	0.99999915	0.21530	0.071589
Z phase	RF	0.99999923	1.5305	0.89434
Z phase	XGBoost	0.99966960	31.751	24.681
Z phase	LightGBM	0.99999991	0.53047	0.31361
Laves	RF	0.99994470	13.957	11.005
Laves	XGBoost	0.99956546	39.126	30.402
Laves	LightGBM	0.99999455	4.3823	3.1837
Scheil solidification interval	RF	0.99980748	0.353572	0.249380
Scheil solidification interval	XGBoost	0.99997118	0.136793	0.089426
Scheil solidification interval	LightGBM	0.99997561	0.125842	0.079232

**Table 5:** Comparison of the mean and median values of the five objectives between the complete dataset and the Pareto set.

Output	Sample Mean	Pareto Mean	Sample Median	Pareto Median
MX	1.0684 × 10^−4^	1.8339 × 10^−4^	0	0
AlN	1.3950 × 10^−4^	4.6659 × 10^−5^	0	0
Z Phase	0.006098	0.005162	0.006381	0.005795
Laves	0.003183	0.001106	0.003154	0.000908
Scheil solidification interval (°C)	224.521	224.612	229.299	226.970

**Table 6:** TOPSIS weighting schemes and corresponding ranking results.

Scheme	Normalized Weights (MX/AlN/Z/Laves/ΔT)	Top 20 Overlap with Scheme A
A: Equal weights	0.2000/0.2000/0.2000/0.2000/0.2000	20
B: Doubled MX weight	0.3333/0.1667/0.1667/0.1667/0.1667	15
C: Increased weights for the three phases	0.1538/0.2308/0.2308/0.2308/0.1538	20
D: Doubled solidification-interval weight	0.1667/0.1667/0.1667/0.1667/0.3333	17

**Table 7:** Compositions of the central reference and five representative candidates (wt.%).

Sample	C	Mn	Cr	Ni	Mo	V	Nb	N	Al	Fe
reference	0.10	0.45	9.00	0.20	0.95	0.21	0.08	0.05	0.01	88.95
1	0.12	0.60	8.00	0.40	0.85	0.24	0.10	0.03	0.00	89.66
2	0.12	0.30	8.00	0.40	0.85	0.24	0.10	0.03	0.00	89.96
3	0.12	0.45	8.00	0.40	0.85	0.24	0.10	0.03	0.00	89.81
4	0.12	0.30	8.50	0.40	0.85	0.24	0.10	0.03	0.00	89.46
5	0.12	0.30	8.00	0.40	0.85	0.21	0.10	0.03	0.02	89.97

**Table 8:** Six output variables for the central reference composition and five representative candidates.

Sample	M_23_C_6_	MX	AlN	Z Phase	Laves	ΔT (°C)
reference	0.022295	0	0	0.006447	3.2623 × 10^−3^	232.216
1	0.025177	7.6060 × 10^−4^	0	0.003869	5.5450 × 10^−5^	255.580
2	0.025195	7.5340 × 10^−4^	0	0.003870	9.4611 × 10^−5^	254.869
3	0.025186	7.5700 × 10^−4^	0	0.003869	7.5162 × 10^−5^	255.235
4	0.025292	7.0361 × 10^−4^	0	0.003880	3.8978 × 10^−4^	251.704
5	0.025388	6.7242 × 10^−4^	0	0.003824	3.9373 × 10^−5^	254.885

## Data Availability

The raw data supporting the conclusions of this article will be made available by the authors on request.

## Supplementary Materials

The following supporting information can be downloaded at: https://www.mdpi.com/article/10.3390/ma19214694/s1. Table S1: Selected hyperparameters of the RF, XGBoost, and LightGBM models for the six thermodynamic descriptors.
